# The Role of Activator Protein-1 (AP-1) Family Members in CD30-Positive Lymphomas

**DOI:** 10.3390/cancers10040093

**Published:** 2018-03-28

**Authors:** Ines Garces de los Fayos Alonso, Huan-Chang Liang, Suzanne D. Turner, Sabine Lagger, Olaf Merkel, Lukas Kenner

**Affiliations:** 1 Ludwig Boltzmann Institute for Cancer Research, 1090 Vienna, Austria; Ines.Garces@lbicr.lbg.ac.at; 2 Unit of Laboratory Animal Pathology, University of Veterinary Medicine Vienna, 1210 Vienna, Austria; Sabine.Lagger@vetmeduni.ac.at; 3 Clinical Institute of Pathology, Medical University of Vienna, 1090 Vienna, Austria; huan-chang.liang@meduniwien.ac.at (H.-C.L.); Olaf.merkel@meduniwien.ac.at (O.M.); 4 Department of Pathology, University of Cambridge, Tennis Court Road, Cambridge CB21QP, UK; sdt36@cam.ac.uk

**Keywords:** Activator Protein-1 (AP-1), Transcription Factors (TFs), Anaplastic Large Cell Lymphoma (ALCL), CD-30, Classical Hodgkin Lymphoma (CHL), Peripheral T-cell Lymphoma (PTCL)

## Abstract

The Activator Protein-1 (AP-1) transcription factor (TF) family, composed of a variety of members including c-JUN, c-FOS and ATF, is involved in mediating many biological processes such as proliferation, differentiation and cell death. Since their discovery, the role of AP-1 TFs in cancer development has been extensively analysed. Multiple in vitro and in vivo studies have highlighted the complexity of these TFs, mainly due to their cell-type specific homo- or hetero-dimerization resulting in diverse transcriptional response profiles. However, as a result of the increasing knowledge of the role of AP-1 TFs in disease, these TFs are being recognized as promising therapeutic targets for various malignancies. In this review, we focus on the impact of deregulated expression of AP-1 TFs in CD30-positive lymphomas including Classical Hodgkin Lymphoma and Anaplastic Large Cell Lymphoma.

## 1. Introduction

### 1.1. AP-1 Transcription Factors

**Figure 1:**
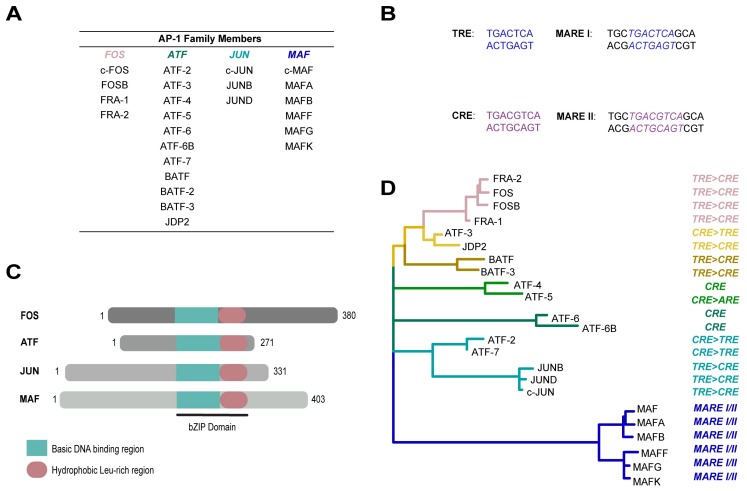
The AP-1 transcription factor family members. (**A**) Table of the different AP-1 transcription factor family subgroups FOS, ATF, JUN and MAF; (**B**) The different DNA response-elements (REs) that AP-1 TFs recognise and to which they bind. The 12-*O*-tetradecanoylphorbol-13-acetate response element (TRE) is the most common; however, depending on dimer configuration, AP-1 TFs can bind to additional elements, such as CRE, MARE I, and MARE II. Note MARE I is an extension of TRE, whereas MARE II is an extension of CRE; (**C**) Schematic representation of the structure of AP-1 proteins including FOS, ATF, JUN, and MAF. AP-1 TFs share two common regions, the basic motif and the leucine zipper; together these regions form the bZIP domain. Sequence data was exported from Nextprot; (**D**) Phylogenetic tree of AP-1 transcription factor family members and their binding REs. The amino acid sequences were aligned with ClustalW implemented with MEGA 7.0.21. The alignment was manually rearranged in Bioedit 7.0.8.0. In order to find the best-fit amino acid substitution model for the alignment, a model test was performed with MEGA 7.0.21. The Maximum Likelihood tree was also calculated with MEGA 7.0.21, applying the parameters obtained from Modeltest.

Transcription factors (TFs) play a crucial role in the maintenance of cellular homeostasis by binding to DNA regulatory sequences thereby modulating the rate of gene transcription. Activator Protein-1 (AP-1) was one of the first mammalian TFs to be identified [1]. Since then, AP-1 has been shown to regulate a wide range of cellular processes, including proliferation, differentiation, and apoptosis [2,3]. AP-1 is a dimeric complex that is composed of members from the JUN (c-JUN, JUNB and JUND), FOS (c-FOS, FOSB, FRA-1 and FRA-2), ATF (ATF-2, ATF-3, ATF-4, ATF-5, ATF-6, ATF-6B, ATF-7, BATF, BATF-2, BATF-3 and JDP2), or MAF (c-MAF, MAFA, MAFB, MAFF, MAFG and MAFK) protein families [2,3] (Figure 1A,D). All active AP-1 members share a common trait: the possession of basic leucine-zipper (bZIP) domains (Figure 1C). Together, the leucine-zipper domain and the adjacent basic domain form a flexible and unique “scissor-shaped” alpha-helical structure [4]. The bZIP domains are essential for dimerization and DNA binding as they recognise different response-elements (REs) in genomic regulatory regions.

The tumour-promoting agent 12-*O*-tetradecanoylphorbol-13-acetate (TPA) activates AP-1 enabling its binding to the TPA-response element (TRE) with the consensus sequence 5′-TGAG/CTCA-3′ [5] (Figure 1B). This TRE element was first discovered in the promoters of metallothionein IIa (hMTIIa) and simian virus 40 (SV40) [6]. Binding of the AP-1 complex to the TRE can also be induced by cytokines, growth factors or onco-proteins, and leads to increased cell survival, proliferation, differentiation and/or transformation. The sequence elements to which AP-1 TFs bind differ depending on the distinctive homo- or hetero-dimer combinations. For instance, JUN/JUN and JUN/FOS recognise the TRE sequence, whereas ATF TFs bind to cAMP response elements (CRE) at the consensus site 5′-TGACGTCA-3′ (Figure 1B) [7]. Interestingly, the crystal structure of MAF family members, bound to their respective DNA binding sites, revealed a unique bZIP structure that enables recognition of a long palindromic sequence referred to as the MAF recognition element (MARE) (Figure 1B) [8]. MARE I is an extension of TRE 5′-TGC-(TRE)-GCA-3′, whereas MARE II is an extension of CRE 5′-TGC-(CRE)-GCA-3′.

JUN proteins can both homo- and hetero-dimerize, whereas FOS proteins only form hetero-dimers, which, particularly for JUN/FOS, are more stable and have greater transcriptional activity [9,10]. Intriguingly, c-FOS and FOSB contain transcriptional activation domains which are lacking in FRA-1 and FRA-2 [2]. Thus, the dimerization partners of JUN influence its role in gene activation and cell cycle regulation [11,12]. For example, JUNB/c-FOS hetero-dimers often dampen transcription, whereas c-JUN/c-FOS heterodimers frequently enhance c-JUN transforming abilities, highlighting the differential effects of the various AP-1 TFs [13]. Furthermore, the effects of JUND on transcription are harder to decipher because this TF has been identified as both an oncogene and a tumour suppressor depending on the cellular context. Most notably, when the tumour suppressor Menin interacts with JUND, it inhibits its transcriptional activity, indicating that JUND might be involved in neoplastic growth suppression [14]. However, when JUND is released from the transcriptional restraint of Menin, JUND switches from a positive to a negative regulator of growth. This transient regulation, resulting in opposite functions, underlines the complexity in deciphering the role of JUND in cellular transformation. Nevertheless, as a general rule, AP-1 TFs can be categorised as “strong activators” of gene transcription (e.g., c-FOS, c-JUN) and “weak trans-activators” (e.g., FRA-1, JUND) [5,11].

At the cellular level, AP-1 TFs regulate cell proliferation by activating or inhibiting the expression of key components of the cell cycle machinery. c-JUN and the FOS protein family members positively regulate cellular proliferation, in comparison to JUNB, which negatively regulates proliferation [15]. c-JUN regulates cell cycle progression in a dual fashion by inhibiting the p53 tumour suppressor and by stimulating cyclin D1 [16]. The reduction in p53, which consecutively leads to dampening of the cyclin-dependent kinase inhibitor (CDKI) p21, accelerates the transition from G1 to S phase [17]. On the other hand, JUNB negatively regulates cell proliferation by inhibiting the progression into S phase via activation of the CDKI p16^INK4a^ and repression of cyclin D1 [18,19]. As JUNB was found to activate tumour suppressor genes, it is commonly referred to as a c-JUN antagonist. However, in recent years, contradicting evidence regarding the tumour suppressive role of c-JUN has emerged. For example, despite having been identified as a potent oncogene and tumour promoter, c-JUN is responsible for inducing apoptosis in UV-exposed cells [20]. Furthermore, c-JUN-deficient cells were found to undergo premature senescence as a result of spontaneous DNA damage, suggesting that c-JUN plays a role in stimulating DNA repair [21].

### 1.2. Transcriptional and Translational Regulation of AP-1

AP-1 TF family members are subject to strict transcriptional, translational and post-translational regulation that are cell type- and context-specific except in the case of malignancies whereby aberrant activity can drive tumour growth.

At the transcriptional level, detailed analyses of the *c-JUN* promoter revealed a high-affinity AP-1 binding site. Mutations in this region abolished the induction of *c-JUN* mRNA [22]. This observation suggests a regulatory feedback loop in which AP-1 activates the *c-JUN* promoter, prolonging its activity and amplifying its expression thereby regulating overall gene expression. One of the few studies directly investigating AP-1 translation rate, revealed that the oncogenic fusion protein Nucleophosmin–Anaplastic Lymphoma Kinase (NPM–ALK), regulates neoplastic transformation by increasing the number of ribosomes bound to *JUNB* mRNA, which in turn renders the translation of JUNB more effective [23].

In addition to transcriptional and translational regulatory mechanisms, AP-1 TFs are subject to a variety of post-translational modifications which affect their activity, stability, localization, and interaction properties. Initial investigations revealed that external stimuli influence the phosphorylation and differential expression patterns of AP-1 proteins [24,25]. For example, c-JUN activation is regulated by Stress Activated Kinases (SAPKs), most commonly referred to as c-JUN *N*-terminal Kinases (JNKs) [26]. Alternative forms of the three JNK proteins (JNK1, JNK2, JNK3) exist, with different abilities to bind and phosphorylate AP-1 family members [27,28]. Five phosphorylation sites have been reported on serine and threonine residues of c-JUN, regulating its trans-activity by distinct mechanisms [29,30]. JNK-stimulated phosphorylation of c-JUN’s *N*-terminal domain at residues S63/S73 facilitates its physical interactions with co-activators, promoting the expression of target genes [31,32], whereas c-JUN phosphorylation on T91/T93 residues releases c-JUN target genes from transcriptional repression [33]. This site-specific phosphorylation regulated by the JNK cascade provides c-JUN with a multi-faceted transcriptional nature. Furthermore, it is interesting to note that phosphorylation of the *C*-terminal end of c-JUN results in its proteasomal degradation [34].

An essential repression mechanism for AP-1 is its rapid degradation by the proteasome [35]. Interestingly, c-FOS is degraded by the proteasome independently of its own ubiquitination marks [36,37], whereas c-JUN is only degraded in an ubiquitin-dependent manner [38]. Recent evidence also suggests that c-JUN/c-FOS dimers are degraded by the SUMO pathway [39,40,41]. Furthermore, reversible protein acetylation is also implicated in AP-1 regulation, as osmotic stress downregulates c-JUN via the histone deacetylase, HDAC3-dependent transcriptional repression [42]. Further investigations revealed that HDAC inhibitors suppress c-JUN binding to *Cyclooxygenase-2* (*COX-2*), *Cyclin D1*, and *Collagenase-1* promoter regions, thereby blocking transcription [43]. More recently, HDAC inhibitors have been reported to transcriptionally suppress both *c-JUN* and *FRA-1* and mechanistically block c-JUN/FRA-1 dimerization, affecting neuroblastoma cell growth [44]. These findings highlight a connection between histone acetylation status and transcriptional activity of AP-1 factors.

MicroRNAs (miRNAs), are small non-coding RNAs of about 19-23 base-pairs that mediate post-transcriptional silencing and also influence AP-1 activity [45]. During early T lymphocyte activation, miRNA-21 is induced, which promotes the Mitogen-Activated Protein Kinase (MAPK)/Extracellular Signal-regulated Kinase (ERK) pathway and JNK signalling and enhances AP-1 activity [46,47]. Similarly, B cell receptor activation induces miRNA-155 expression via a conserved AP-1 element [48]. It is thus critical to investigate the dose-dependent activity of specific miRNAs and AP-1 members in selective cellular environments to yield future therapeutic strategies.

In summary, AP-1 TFs are regulated by dimer configuration, gene transcription, post-translational modifications and protein interactions [2]. Despite large efforts, the physiological functions of AP-1 still remain to be elucidated, mostly because of the multi-step complexity of regulation of their activity and their tissue-specific functionality.

### 1.3. AP-1 Functions in Tumourigenesis

c-JUN and c-FOS were initially identified as retroviral onco-proteins (v-Jun and v-Fos) of the Avian sarcoma virus 17 (ASV17) and Finkel–Biskis–Jinkins murine sarcoma virus, respectively [49,50]. Activation of the mammalian AP-1 counterparts of the viral proteins was shown to lead to cellular transformation and oncogenesis. Genetic manipulation of JUN and FOS proteins in mice have highlighted the critical and selective role of AP-1 TFs in development and tumour formation [51].

When deregulated, either by overexpression or downregulation, AP-1 factors promote tumourigenesis depending on the cellular context. In addition to cell-autonomous oncogenic capacities, AP-1 TFs were suggested to act as mediators of oncogenic transformation via growth factors (e.g., Hepatocyte growth factor (HGF) [52]), onco-proteins (e.g., Tumour Necrosis Factor alpha (TNF-α) [53]), or cytokines (e.g., interleukin-1 (IL-1) [54]), altogether supporting cell proliferation, growth and survival. Similarly, AP-1 TFs interact with hypoxia-inducible factor 1 alpha (HIF1a), establishing a link between AP-1 and angiogenesis [55]. Multiple studies have therefore highlighted the implication of AP-1 TFs in major cancer-related pathways, including inflammation, differentiation, cellular migration, metastasis, angiogenesis and wound healing [3].

AP-1 TFs are deregulated in both solid tumours and haematological malignancies. In this review, we will present the current literature on the role AP-1 TFs play in lymphoid malignancies, focusing on CD30-positive lymphomas, specifically, Classical Hodgkin Lymphoma (CHL) and the Non-Hodgkin Lymphoma (NHL) sub-type peripheral T-cell lymphoma (PTCL) which constitutes a heterogeneous group of disease entities often associated with a poor prognosis [56,57,58,59]. The World Health Organisation classifies CHL and PTCL into sub-groups based on the presentation of the lymphoma and their clinical features [60,61,62] (Table 1).

## 2. AP-1 TFs in Classical Hodgkin Lymphoma (CHL)

### 2.1. Deregulated Signalling Pathways and AP-1 TFs in CHL

HL is a nodal lymphoproliferative disorder characterised by dysplastic giant cells, named Hodgkin and Reed–Sternberg cells (HRS) and driven by clonal, neoplastic cells of a B cell origin [63,64]. Numerous signalling pathways are deregulated in HRS, including Nuclear Factor-κB (NF-κB), Janus Kinase-Signal Transducer and Activator of Transcription (JAK–STAT), IFN regulatory factor (IRF) [65] and Phosphoinositide 3-Kinase (PI3K)–Protein Kinase B (AKT) [66].

#### 2.1.1. The NF-κB/AP-1 Signalling Axis in CHL

NF-κB activation in HRS cells has been linked to alterations in the NF-κB inhibitor IκB. Under homeostatic conditions, phosphorylation of IκB leads to its degradation, which in turn releases NF-κB subunits into the nucleus where they activate the transcription of target proteins [67]. Mutations in IκB and/or constitutive activity of kinase receptors upstream of the signalling pathway result in NF-κB insensitivity to IκB [68,69]. Furthermore, overexpression of a dominant negative IκB results in decreased nuclear NF-κB activity in HRS cells via dampening of proliferation and enhancement of apoptosis [68]. As such, the NF-κB signalling pathway is constitutively activated in HRS cells, driving their uncontrolled proliferation and survival partly via JUNB [68,69,70,71].

Furthermore, elevated levels of pro-inflammatory cytokines and chemokines, such as IL-6, a pro-inflammatory cytokine involved in haematopoiesis [72], and CXCL8, a chemokine responsible for the recruitment and activation of immune cells [73], maintain malignant proliferation. Additionally, continuous production of IL-6 and CXCL8 results in the enhanced survival of lymphocytes, which further increases serum cytokine/chemokine levels [74,75]. Finally, the identification of both AP-1 and NF-κB binding sites in the promoter region of *IL-6* and *CXCL8* cemented the NF-κB/AP-1/IL-6/CXCL8 axis [24,76,77]. In addition, NF-κB and AP-1 TFs share common mechanisms of activation as they appear to be simultaneously activated by the same stimuli [78,79]. For example, JNK activation via inflammatory or stress-related cytokines results in the phosphorylation of JUN and the nuclear translocation of NF-κB [80]. This is supported by the fact that many genes require the concomitant activation of AP-1 and NF-κB, explaining the shared stimuli resulting in their activation and cooperative nature [79,81]. Furthermore, the response of AP-1 TFs is strikingly enhanced when NF-κB subunits are present, as is highlighted by the p65 subunit of NF-κB which acts as an accessory protein for AP-1 TF dimerization [82]. In addition, the expression of the pro-inflammatory TF IRF5, together with NF-κB, activates AP-1, inducing the expression of cytokines and chemokines [65]. Overall, this suggests that NF-κB can either directly or indirectly, via AP-1 TFs, control HRS cell growth.

#### 2.1.2. The STAT/AP-1 Signalling Axis in CHL

The JAK/STAT pathway is commonly involved in maintaining elevated proliferation of malignant cells by interacting with TFs such as the AP-1 family members [83,84]. The TFs STAT3, STAT5A/STAT5B and STAT6 are all elevated in HRS cells and are involved in mediating cellular transformation and expression of the diagnostic marker CD30 [85,86,87]. The overexpression of constitutively active forms of either STAT5A or STAT5B in normal B cells leads to upregulation of the Tumour Necrosis Factor Receptor Superfamily 8 (TNFRSF8), also known as CD30, a cell membrane protein normally expressed by activated T and B cells [85].

**Figure 2:**
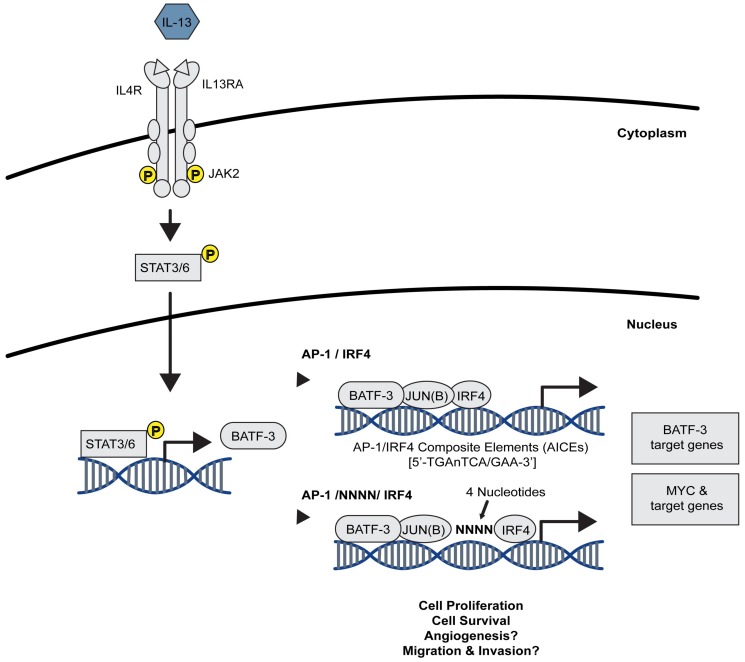
The described JAK/STAT/BATF/MYC signalling cascade in Classical Hodgkin Lymphoma (CHL) and Anaplastic Large Cell Lymphoma (ALCL). JAK2 activation, via IL-13 stimulation, results in the phosphorylation (yellow) of STAT3/6. STAT3/6 translocates into the nucleus where it promotes the expression of *BATF-3*, which dimerizes with c-JUN or JUNB to form stable and active AP-1 TFs. BATF-3/c-JUN dimers directly bind to the *MYC* gene promoter resulting in an increase in MYC and BATF-3 target gene expression, promoting proliferation and survival in CHL and ALCL. Figure adapted from [88].

Whilst STAT5A/5B have been associated with CD30 expression, JAK2 and STAT3/6 orchestrate BATF-3 expression in HRS cells by binding to STAT consensus sequences in its promoter [88]. BATF-3 is highly expressed in CD30^+^ CHL, although it relies on additional AP-1 cofactors to dimerize, as BATF family members lack a transcriptional activation domain [89,90,91]. Indeed, co-immunoprecipitation and mass spectrometry revealed that BATF-3 directly interacts with JUN and JUNB in HRS cells, which in turn activates *MYC* transcription by directly binding to its promoter [88,92]. This novel oncogenic STAT–BATF-3/JUN–MYC signalling axis seems to be essential for HRS cell survival (Figure 2) [88]. Overall, these data indicate that general deregulation of the JAK/STAT signalling pathway, its upstream regulators and downstream effectors promote CHL progression.

### 2.2. Deciphering the Cross-Talk between AP-1 and Cell Surface Proteins in CHL

**Figure 3:**
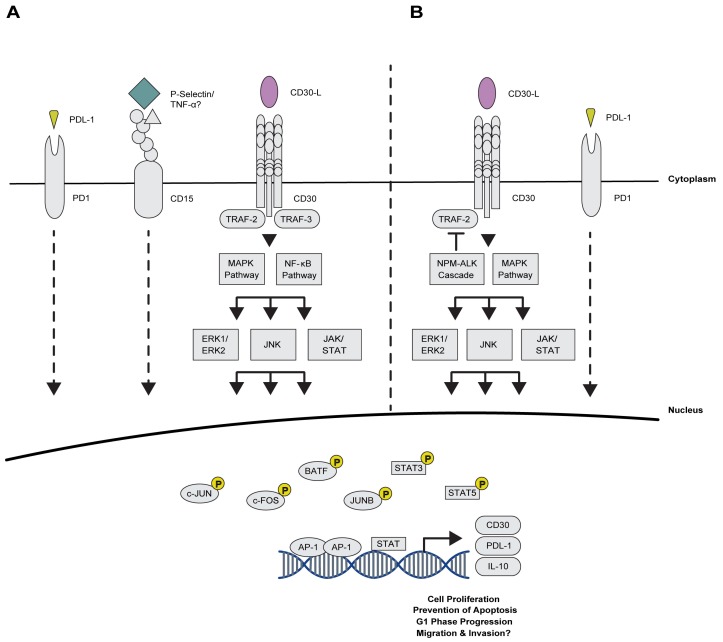
The intricate relationship between CD30 and AP-1 in Classical Hodgkin Lymphoma (CHL) and Anaplastic Large Cell Lymphoma (ALCL). (**A**) Cell surface protein signalling involved in CHL. In CHL, the PD-L1 enhancer binds AP-1 TFs resulting in an increase in *PD-L1* promoter activity. Similarly, binding of P-Selectin (green) to CD15 (Lewis X) induces the translocation of c-JUN into the nucleus and an increase in AP-1 DNA binding activity. CD30 activation, mediated via CD30-ligand (CD30-L) (purple), results in the formation of a homo-trimer. The receptor, which lacks a kinase domain, recruits TNFR-associated factors (TRAF), which activate NF-κB and MAPK/ERK downstream signalling pathways. The activation of NF-κB and MAPK/ERK results in the phosphorylation of AP-1 and STAT TFs. Following activation, JUNB translocates to the nucleus where it binds to the unmethylated *CD30* promoter and maintains elevated CD30 levels; (**B**) In ALCL, Nucleophosmin-Anaplastic Lymphoma Kinase (NPM–ALK) controls CD30 expression via the phosphorylation of downstream targets, such as STAT and AP-1. Once activated, the STATs and AP-1 complexes cooperate to enhance CD30 transcription, fuelling a positive feedback loop. Thus, when activated in ALCL, CD30 stimulates the activation of both the canonical and alternative NF-κB pathways in addition to indirectly stimulating its upregulation. Additionally, NPM–ALK is believed to upregulate PD-L1 directly via phosphorylation of STAT and AP-1 TFs, or indirectly via the induction of interleukin-10 (IL-10), which in turn is known to activate the JAK/STAT signalling pathway.

CD proteins are expressed on the surface of a variety of cells including lymphocytes and leukocytes of the immune system [93]. CD15 and the TNFRS members CD30, CD40, and CD95 are expressed on HRS cells. The specific expression of these proteins provides a tool for CHL diagnosis. Additionally, this characteristic cell surface marker expression profile suggests the possible involvement of CD15 and CD30 in the pathogenesis of CHL by activating downstream signalling pathways such as NF-κB (Figure 3) [69].

#### 2.2.1. Maintaining Proliferation via AP-1 and the TNFRS Family Members

CD30 signalling activates NF-κB and MAPK/ERK pathways, suggesting that it plays a role in cellular proliferation and anti-apoptotic signalling [94,95,96,97]. Interestingly, activation of the NF-κB and MAPK/ERK signalling cascades can be directed either by ligand-dependent or ligand-independent CD30 [96,97,98,99]. Subsequently, Watanabe et al. hypothesised that polymorphic changes affecting the length of CD30 microsatellite sequences (MS) may result in weakening of transcriptional repression culminating in the overexpression of CD30 in HRS cells [100,101]. Furthermore, the discovery of an AP-1 site in the CD30 MS suggested that relief of suppression of the CD30 promoter is mediated via JUNB [100]. Additional investigations revealed that enhanced JUNB protein expression acts on the unmethylated CD30 promoter to maintain elevated CD30 levels in CHL [96]. More recently, Watanabe et al. localized a cis-acting enhancer in the JUNB promoter that is regulated by E26 transformation-specific-1 (Ets-1). The study suggests that Ets-1 enhances JUNB promoter activation in a CD30-dependent fashion, and knock-down of Ets-1 dampens the expression of both JUNB and CD30 [101]. Cumulatively, these findings suggest a critical role for CD30/AP-1 signalling in maintaining malignant HRS cells.

Similar to CD30, the cell surface oligosaccharide moiety CD15 is expressed on malignant HRS cells [102]. Despite a lack of understanding of the contribution of CD15 expression to the pathobiology of CHL, this antigen serves as a diagnostic marker [103]. While studying the contribution of CD15 to cell adhesion, Ohana et al. reported that binding of the CD15 antigen, induced nuclear translocation of c-JUN and a significant increase in AP-1 DNA binding activity [104]. As c-JUN appears to be a crucial mediator in propagating and maintaining a malignant phenotype, this study suggests that these interactions are significant for enhancing tumour–stroma interaction, adhesion and metastasis.

#### 2.2.2. Novel Association of AP-1 with Immuno-Surveillance and -Suppression Mechanisms

Programmed death 1 (PD-1) is a critical co-inhibitory molecule that regulates tumour immune escape and inhibits T cell receptor signalling [105]. In recent years, PD-1 and its ligands, PD-L1 and PD-L2, have been detected in a variety of tumour types [105]. Recently, it was discovered that HRS cells enhance PD-1 signalling (Figure 3) [106,107,108]. Further investigations revealed that the PD-L1 enhancer binds AP-1 TFs resulting in an increase in PD-L1 promoter activity. Interestingly, PD-L2 transcription is not enhanced in an AP-1-dependent manner even though PD-L2 is also overexpressed in HRS cells [109]. These findings suggest a potential AP-1/PD-1 axis which could be exploited as a future therapeutic option for CHL, especially since PD-1 and its ligands have already highlighted the clinical efficacy of targeted immunotherapy [105].

## 3. Involvement of AP-1 TFs in the Pathogenesis of CD30^+^ Peripheral T-Cell Lymphomas (PTCLs)

### 3.1. Anaplastic Large Cell Lymphoma (ALCL)

ALCL, an aggressive CD30^+^ lymphoma which accounts for 12.1% of all PTCL cases [110], is divided into two entities on the basis of the presence or absence of the receptor tyrosine kinase Anaplastic Lymphoma Kinase (ALK) (ALCL, ALK^+^ or ALCL, ALK^−^). In 70% of ALK^+^ cases of ALCL, the t(2;5)(p23;q35) chromosomal translocation drives the progression of the disease. The translocation breakpoint encodes the C-terminal part of *Nucleophosmin* (*NPM*) and the kinase domain of *ALK*. In the remaining 30% of ALK^+^ ALCL patients, other ALK fusion partners have been described (e.g., *TPM3*, *TPM4*, *TFG*, *ATIC*, *CLTC*, *MSN*, *MYH9* and *ALO17*) [62,111,112,113,114]. Whilst the oncogenic activity of these ALK fusion proteins is responsible for disease progression in ALK^+^ ALCL cases, the pathogenesis of ALK− ALCL cases remains unclear [115,116,117].

#### 3.1.1. AP-1 TFs Are Expressed in ALCL, Mediate Key Signalling Pathways and Account for Typical Features of this Malignancy

Constitutive activation of the AP-1 TF family members *FRA-2*, *JUNB*, *JUN*, *ATF-3* and *BATF-3* is observed in nearly all ALCL cases, regardless of the ALK status [71,118,119,120], whereas genomic gains of the *FRA-2* and *JUNB* loci are described in some ALCL cases [121]. AP-1 TFs are activated as a consequence of NPM–ALK activity downstream of the MAP/SAPK pathways [122,123,124]. In particular, *JUNB* or c-JUN expression protects cells from apoptosis, thereby enhancing cellular proliferation and colony formation [23,71,121,123,125]. Given the activity of c-JUN, JUNB, FRA-2, and ATF-3 in ALCL, it is likely that other AP-1 activating factors play a role in lymphomagenesis [92,118].

In addition to MAP/SAPK pathways, many other signalling pathways are involved in AP-1 signal transduction in ALCL. For example, p-STAT3 binds to the three STAT binding sites in the promoter of *BATF-3*, and, therefore, BATF-3 expression is regulated at least in part by JAK/STAT signalling [88]. In turn, BATF-3, together with JUN family proteins and IRF4, binds to AP-1/IRF4 composite elements (AICEs) present in the promoter region of *MYC* as well as of various other target genes (Figure 2) [88,92,126]. Additionally, JUNB is associated with the mechanistic Target of Rapamycin (mTOR) pathway in ALK^+^ ALCL but not in ALK^−^ ALCL, as downregulation of JUNB expression is observed after rapamycin treatment [23]. As such, AP-1 TFs are central to the proliferation and survival of ALCL, mediating several signalling pathways.

As well as playing a functional role in the activation of oncogenic pathways and dysregulation of cellular proliferation, AP-1 TFs account for some key features of ALCL such as the expression of CD30, a diagnostic marker of this malignancy. Cell surface CD30 expression in ALCL is mediated via ALK-induced activation of JUNB via ERK1/2 and MAPK. Following its activation, JUNB binds the AP-1 site in the upstream promoter region of *CD30* (Figure 3) [96,127]. Interestingly, ALCL and CHL, both CD30-expressing malignancies, share a similar expression pattern of *FRA-2*, *JUNB*, *JUN* and *ATF-3*, whereas high expression of *BATF-3* is specific to ALCL [71,118,119,120,124].

Another feature of ALCL, potentially in part attributable to AP-1 activity, is the absence of a cell surface T cell receptor (TCR). Whilst molecular TCR rearrangements are seen in ALCL, the TCR is not expressed on the surface of ALCL cells [128]. In addition, TCR proximal signalling proteins such as ZAP-70, CD3ε, and LAT are actively suppressed via transcriptional and epigenetic mechanisms in an ALK-dependent manner [128,129,130]. Turner et al. demonstrated that NPM–ALK, via the RAS/MAPK pathway and calcium signalling, induces transcription via AP-1/NFAT composite binding sites, mimicking activated TCR signalling [124].

#### 3.1.2. AP-1 TFs Provide Therapeutic Targets for the Treatment of ALCL

**Figure 4:**
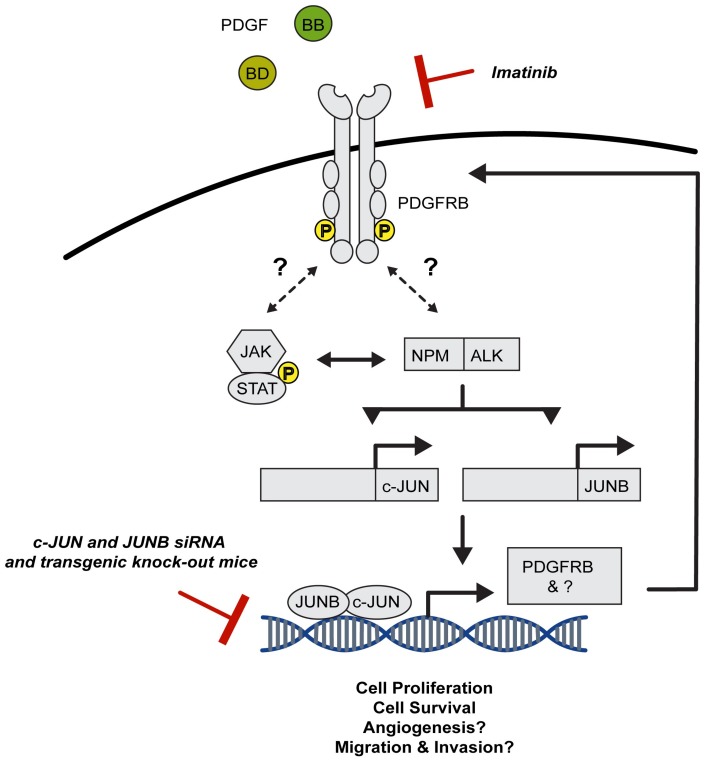
Blockade of the PDGFRB/STAT/NPM-ALK/JUN signalling cascade in Anaplastic Large Cell Lymphoma (ALCL). Stimulation via PDGF-BB or –DD results in the dimerization and trans-phosphorylation of PDGFRB. The receptor phosphorylates the JAK/STAT signalling cascade, which is known to interact with the oncogenic NPM–ALK fusion. The downstream effector targets of NPM–ALK, c-JUN and JUNB, directly bind to the *Pdgfrb* promoter resulting in an increase in PDGF expression, overall propagating a malignant signalling cascade. Treatment with Imatinib, a tyrosine kinase inhibitor, results in a decrease in tumour burden and increased survival of c-JUN and JUNB transgenic knock-out mice harbouring the constitutively active NPM–ALK fusion in CD4^+^ T cells [131]. Targeting PDGFRB or TFs that regulate PDGFRB expression is thus a rational and effective therapy for NPM–ALK-driven lymphomas.

Deletion of both *JUN* and *JUNB*, but neither alone, impairs NPM–ALK-driven lymphomagenesis in an ALCL mouse model expressing the human *NPM–ALK* transgene under the *CD4* promoter. The survival of *CD4*-NPM–ALK mice with both *JUN* and *JUNB* deletions in their T cells (*CD4*-NPM–ALK^CD4ΔJUN/JUNB^) is substantially prolonged in comparison with that of wild-type *CD4*-NPM–ALK mice and accompanied by reduced proliferation and significantly increased apoptosis [131]. TFs are notoriously difficult to specifically inhibit, whereas the protein products of their activity, such as Platelet Derived Growth Factor Receptor Beta (PDGFRB), are easier to target. PDGFRB is a type III tyrosine kinase receptor, whose expression is absent in *CD4*-NPM–ALK^CD4ΔJUN/JUNB^ mice as a result of the abrogation of JUN binding to the AP-1 site in its promoter. This murine model predicts that inhibitors of PDGFR such as Imatinib, an inhibitor of BCR–ABL kinase, receptor tyrosine kinase KIT and PDGFRB, may provide a viable therapeutic approach. Indeed, a dramatic increase in overall survival was observed in Imatinib-treated *CD4*-NPM–ALK transgenic mice (Figure 4).

### 3.2. Involvement of AP-1 TFs in PTCL-Not Otherwise Specified (PTCL-NOS)

PTCL-NOS represents the largest PTCL entity accounting for 25.9% of all cases and is characterised by pronounced immunophenotypic and morphological heterogeneity as well as an absence of defining molecular criteria [62,110]. However, two subtypes with distinct oncogenic pathway activation and prognosis have recently been identified, with expression of the transcription factors *TBX21*/*EOMES* or *GATA3* and their target genes [132]. On the basis of the imposed cut off of the immunohistoscore, CD30 expression is detected in 14–52% of PTCL-NOS cases [133,134]. CD30 signalling activates the NF-κB and MAPK/ERK pathways leading to enhanced JUNB expression in PTCL. JUNB in turn activates CD30 transcription resulting in a positive feedback loop [135]. Furthermore, the expression of c-JUN can only be detected in CD30^+^ (not in CD30^−^) PTCL-NOS [136]. Indeed, unsupervised transcriptome clustering of PTCL-NOS correlates with CD30 expression: the CD30-positive PTCL-NOS group features high expression of the transcription factors JUNB and MUM1/IRF4, whereas these are largely absent in the majority of CD30-negative cases. Conversely, several proteins involved in TCR signalling (e.g., tyrosine kinases LCK, FYN and ITK), T-cell differentiation/activation (e.g., CD69, CD52 and ICOS) and the transcription factor NFATc2 are mainly expressed in CD30-negative cases. Accordingly, in ALCL, suppression of *LCK*, *FYN*, *ITK*, *CD69*, *CD52*, *ICOS* and *NFATc2*, in addition to induction of *JUNB* and *MUM1*/*IRF4* transcription is also observed [126,135].

### 3.3. Implication of AP-1 TFs in Extranodal, Cutaneous T-Cell Lymphomas (CTCLs)

Similar to other CD30^+^ lymphomas, CTCLs, including cutaneous ALCL and Lymphomatoid Papulosis (LyP), show abnormal JUNB expression which correlates with both CD30 and MAPK/ERK pathway activity [137,138]. However, the exact signalling axes responsible remain to be fully elucidated. To date, most studies have focussed on immunohistochemical findings, although genomic amplification of *JUNB* with concomitant increased expression, leads to deregulated AP-1 activity in CTCL [138]. Furthermore, phosphorylated ERK 1/2 levels correlate with JUNB-expression, suggesting that deregulated JUNB and MAPK activities are responsible for the malignant progression of CTCL [138].

As well as JUNB, other AP-1 TFs have been implicated in the pathogenesis of CTCL. For example, JUND and FRA-2 upregulate the expression of the commonly expressed C-C chemokine receptor 4 (CCR4). An in depth expression analysis of primary CTCL identified the oncogenic cascade FRA-2/JUND–CCR4–MDM2 in skin lesions [122]. siRNA-mediated inhibition of either FRA-2 or JUND expression resulted in a decrease in cell proliferation and a dampening of CCR4 and MDM2 expression in CTCL cell lines [122]. The discovery of CCR4 as a downstream target of FRA-2 and JUND could in the future be exploited, being a tumour-associated antigen in adult T-cell leukaemia/lymphoma (ATLL) and CTCLs [122].

### 3.4. AP-1 in Other PTCLs and Diffuse Large B-Cell Lymphoma (DLBCL)

Angioimmunoblastic T-cell lymphoma (AITL) is an aggressive lymphoma derived from follicular helper T cells and is the second largest category accounting for 18.5% of PTCL cases [110,139]. Activating mutations involved in the AP-1/MAPK pathway were detected in a fraction of AITL cases: three missense mutations of *KRAS* (G13D, A18D, and I36M) and two missense mutations of *STAT3* (E616G and E616K), all of which result in constitutive activation of the AP-1 transcription factor c-FOS [140]. On the other hand, in primary adult T-cell leukaemia/lymphoma (ATLL), strong constitutive activation of NF-κB and AP-1 was also identified [141,142]. Several reports have shown that both ATLL cell lines and primary cells frequently express high levels of *CCR4* and several AP-1 family members, including *FRA-2*, *JUNB*, *JUND*, and *ATF-3*. Except for *ATF-3*, these properties are shared with CCR4-expressing CTCLs as mentioned before [143,144,145,146,147,148,149]. Natural killer (NK) and NK-like T-cell lymphomas represent less than 10% of PTCL cases [61,110]. Genomic imbalances were observed in all studied cases, with gains of *JUNB* in 75% of malignancies examined [121], suggesting that JUNB contributes to lymphomagenesis. Enteropathy-associated T-cell lymphoma and hepatosplenic γδ T-cell lymphoma are extremely rare entities [62,110], which may account for the lack of literature describing the importance of AP-1 factors in these diseases [150,151].

On the basis of the gene expression pattern, diffuse large B-cell lymphoma (DLBCL) is divided into two main entities: the activated B-cell (ABC) and the germinal centre B-cell phenotypes (GCB). It has recently been shown that the more aggressive ABC type cell lines express higher levels of c-JUN, JUNB and JUND [152]. This finding is in agreement with another study using immunohistochemistry in primary patient tissue, which showed a positive correlation between the expression of JUN family members and proliferation markers [153]. Additionally, a screen for c-JUN and phosphorylated c-JUN of 344 CD30-positive T- or B-cell lymphomas, including 11 CD30-positive DLBCL cases, detected a striking link between CD30 and c-JUN expression [136].

## 4. AP-1 and Future Treatment Options

Members of the AP-1 TF family may represent novel therapeutic targets as their involvement in a variety of pathologies, ranging from inflammation to cancer, including atherosclerosis, hepatitis, cardiovascular disease and Parkinson’s disease, has driven research in this area [154,155,156]. Traditionally, therapeutic targeting of TFs has proven difficult. In particular, the high diversity and tissue-specific functions of AP-1 TFs represents a challenge for the development of efficacious drugs that can be used in the clinic. However, recent progress has been made in targeting TFs through novel chemistries and protein structure-guided design strategies [157,158]. In addition, the utilisation of stapled peptides to disrupt protein–protein interactions has proven successful [159].

Several core molecular scaffolds have been found to be associated with anti-AP-1 properties. For instance, SP100030 was the first group of inhibitors of AP-1 and NF-κB transcription activation in Jurkat cells [160,161]. It also showed an inhibitory effect on IL-2/IL-8 production, CD8-positive T-cells and Th1/Th2 cytokine mRNA expression in animal models for inflammatory disorders [161,162]. SPC-389 is another AP-1 and NF-κB inhibitor; however, it was identified to be more selective towards inhibiting AP-1-mediated transcription activity over NF-κB in vitro [163,164,165]. Moreover, one of the most promising AP-1 inhibitors, T-5224, which underwent testing for the treatment of rheumatoid arthritis in a phase II clinical trial was developed by Toyama Chemical Co., Ltd. (Tokyo, Japan) and Kitasato University, Japan [156]. However, in 2008, the company ceased this programme of research for unreported reasons. T-5224 selectively targets the c-FOS subunit of AP-1 without affecting other TFs, e.g., NF-κB/p65, C/EBPα and ATF-2 [156,166]. Recent preclinical studies in mice have shown that this inhibitor reduces lymph node metastasis of oral cancer and targets stem cells in squamous cell carcinoma when combined with cisplatin, suggesting it may be efficacious in the treatment of some cancers [167,168]. Several other preclinical compounds have also been developed, although their clinical usefulness is hampered by low specificity, high IC_50_ values in the µM range, or a lack of oral bioavailability [160,164,169]. Despite the tremendous efforts researchers have put into high-throughput screening, hit-to-lead optimisation and protein crystallography to improve drug properties, only one selective AP-1 inhibitor has entered human clinical trial so far. Many of the currently available compounds still lack specificity and also target other TFs including NF-κB. Hence, in order to achieve a viable therapeutic strategy targeting AP-1, an urgent need for developing more potent, specific and efficacious inhibitors which can be used in the clinic still remains.

Apart from directly targeting AP-1 members, downstream targets of AP-1 may also serve as potential therapeutic targets. For example, we have shown that *JUN*/*JUNB* AP-1 hetero-dimers bind to the promoter of *Pdgfrb* and regulate its expression [131]. As mentioned previously, we showed that treatment of tumour-bearing mice with Imatinib, a PDGFR kinase inhibitor, resulted in reduced tumour growth and a significant increase in overall survival [131]. In addition, treatment of a refractory, late-stage NPM–ALK^+^ ALCL patient with Imatinib led to complete and sustained remission [131]. These findings suggest that targeting PDGFRB, an AP-1 downstream target, is a promising alternative therapeutic option for ALCL, which is currently being explored in a clinical trial (EudraCT Nr.: 2013-003505-26) (Figure 4).

## 5. Conclusions

AP-1 is a pivotal homo- or hetero-dimeric TF family involved in a wide range of cellular processes including proliferation, differentiation and apoptosis. In cancer development, AP-1 acts as a double-edged sword with both oncogenic and tumour suppressive activities, rendering the functional characterisation of these TFs challenging. However, recent studies have shed light on the involvement and role of AP-1 TFs in lymphoid malignancies [92,126,131]. In general, c-JUN acts as an oncogenic driver, whereas JUNB and JUND have tumour suppressive effects. AP-1 family members are involved in a variety of mitogenic signalling pathways such as RAS/MAPK, PI3K/AKT/mTOR and JAK/STAT/MYC [23,88,124,170]. As such, it is unsurprising that they are exploited by cancer cells to drive tumourigenic processes, in particular in CD30^+^ lymphomas. Hence, they represent key targets for therapeutic intervention in this class of diseases.

## Figures and Tables

**Table 1:** Table of lymphoproliferative disorders. Lymphoid neoplasms were sub-grouped according to the World Health Organisation 2016 classification [62].

Cancer Family	Neoplasms	Major Groups	Sub-Groups
Lymphoproliferative Disorders [62]	Hodgkin Lymphoma (HL) [63,64]	Classical Hodgkin Lymphoma (CHL) [64]	
	Peripheral T-cell Lymphoma/Non-Hodgkin Lymphoma (NHL) [56,57,58,59,60,61,62]	Nodal Lymphoma [56,57,62]	Anaplastic Large Cell Lymphoma (ALCL)
			Angioimmunoblastic T-cell Lymphoma (AITL)
			PTCL-Not Otherwise Specified (PTCL-NOS)
		Extranodal Lymphoma [58,59,62]	Enteropathy-associated T-cell Lymphoma (EATL)
			Hepatosplenic Υδ T-cell Lymphoma
			Natural Killer (NK)/NK—like T-cell Lymphoma
		Extranodal-cutaneous Lymphoma [58,60,62]	B-cell Cutaneous Lymphoma
			T-cell Cutaneous Lymphoma
			NK-cell Cutaneous Lymphoma
		Leukaemic and Disseminated Disease [61,62]	Adult T-cell Leukaemia/Lymphoma (ATLL)
